# Clinical Characteristics and Risk Factors for Evisceration in Trauma-Dominant Orbital Cellulitis: A 10-Year Review

**DOI:** 10.3389/fmed.2022.935022

**Published:** 2022-06-17

**Authors:** Zhaoxin Jiang, Xueying Zhongliu, Xiaohu Ding, Yao Yang, Fang Duan, Xiaofeng Lin

**Affiliations:** State Key Laboratory of Ophthalmology, Guangdong Provincial Key Laboratory of Ophthalmology and Visual Science, Zhongshan Ophthalmic Center, Sun Yat-sen University, Guangzhou, China

**Keywords:** orbital cellulitis, clinical characteristics, risk factors, trauma, evisceration

## Abstract

**Purpose:**

To analyze the clinical characteristics of trauma-dominant orbital cellulitis (OC) and evaluate the risk factors associated with evisceration.

**Methods:**

This retrospective study included inpatients diagnosed with orbital cellulitis at the Zhongshan Ophthalmic Center between January 2010 and December 2020. The demographic features, etiology, clinical characteristics, microbiological isolates, and risk factors associated with evisceration were analyzed.

**Results:**

Among 148 consecutive subjects (*n* = 148, 148 eyes), the mean age was 42.07 ± 20.35 years and 70.27% were male. Penetrating globe injuries were the most common (52.03%). No light perception (NLP) was recorded in 50% of patients on admission. Endophthalmitis was observed in 103 cases (69.59%), intraocular foreign bodies (IOFB) were detected in 43 cases (29.05%), and total corneal melting was observed in 31 cases (20.95%). Sixty patients (40.54%) underwent evisceration. Logistic regression analysis showed that total corneal dissolution [odds ratio (OR) = 83.019, *P* = 0.000], IOFB (OR = 3.402, *P* = 0.016), and NLP (OR = 0.185, *P* = 0.001) were risk factors for evisceration. Microorganism detection showed that *Pseudomonas aeruginosa* and *Bacillus cereus* were the leading pathogens.

**Conclusion:**

Among hospitalized trauma-dominant OC patients, middle-aged men were the major subjects and penetrating globe injury was the major cause. Significant complications such as complete visual loss and evisceration were unavoidable in many patients with OC in the current study. NLP, IOFB, and total corneal melting were the risk factors for evisceration.

## Introduction

Orbital cellulitis (OC) is an uncommon inflammation that involves tissues located posterior to the orbital septum within the bony orbit (1, 2). It can be caused by the spread of infection from either extension from the periorbital structures (most commonly the adjacent ethmoid or frontal sinuses), intraorbital infection (endophthalmitis or dacryoadenitis), blood (bacteremia with septic emboli), or exogenous causes (trauma or foreign bodies) (1). Although rare, OC causes important complications including vision loss, endophthalmitis, and cavernous sinus thrombophlebitis (3, 4). In developed countries, secondary infections extending from the paranasal sinuses are the most frequent predisposing factors (5, 6). The clinical characteristics, categories, and surgical drainage criteria for sinusitis-related OC have recently been reported (7–9).

However, ocular trauma is an increasingly common cause of OC in developing regions and may present completely different characteristics from reports based on sinusitis-dominant OC patients. For instance, a relatively good prognosis in vision has been reported in sinusitis-dominant OC, in which abscess drainage is the major surgery (10). In contrast, in our clinical practice, a high proportion of patients with OC have no light perception (NLP) at admission and require evisceration surgery. Besides, ocular trauma is an important cause of endophthalmitis. Exogenous endophthalmitis occurs when infecting organisms enter the eye *via* direct inoculation, such as in open globe injury or intraocular surgery. Endogenous endophthalmitis is less common and results from hematological spread from a distant infection (11). To our knowledge, few studies have analyzed the characteristics of trauma-dominant OC; moreover, the risk factors associated with evisceration remain unclear.

We previously identified ocular trauma and intraocular foreign bodies (IOFB) as important risk factors for endophthalmitis (11, 12) and summarized the microbiological isolates and antibiotic susceptibilities of culture-proven endophthalmitis (13, 14). In the present study, we reviewed the data of patients hospitalized with OC in the past 10 years to (1) describe the clinical characteristics of OC, (2) explore the microbiological isolates of OC, and (3) identify the risk factors related to NLP and evisceration in patients with OC.

## Materials and Methods

### Population

This study was performed in accordance with the tenets of the Declaration of Helsinki and was approved by the Medical Ethics Committee of Zhongshan Ophthalmic Center, Sun Yat-sen University. The clinical records of consecutive patients admitted with OC between January 2010 and December 2020 at the Zhongshan Ophthalmic Center were retrospectively reviewed. Written informed consent to participate in this study was provided by the participants’ legal guardians or next of kin. Written informed consent was obtained from each individual(s) for the publication of potentially identifiable images or data included in this article.

### Definitions

**FIGURE 1:**
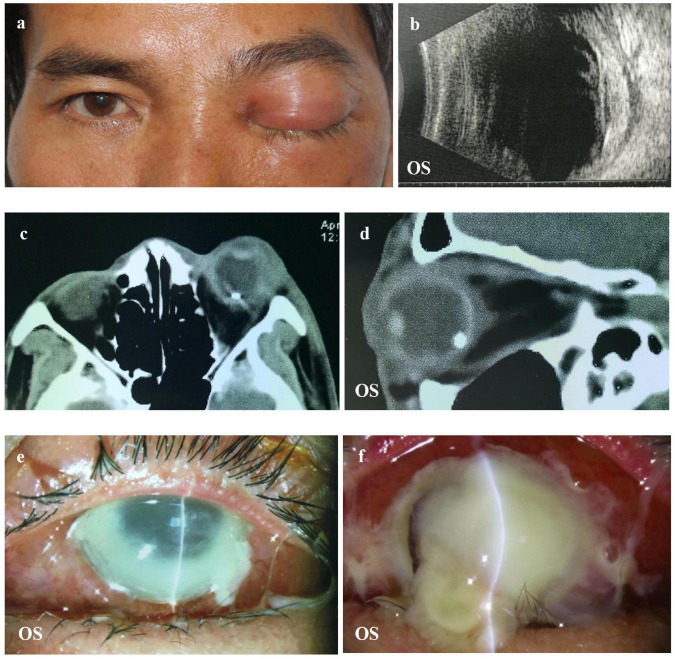
Representative image of a patient with OC. **(a,b)** The patient complained of vision loss and orbital pain 1 day after a foreign body splashed into the left eye. B-scan showed increased choroidal thickness. **(c,d)** CT showing inflammation of the orbital tissue and IOFB in the left eye. **(e,f)** Uncontrolled total corneal melting that required an evisceration. OC, orbital cellulitis; CT, computed tomography; IOFB, intraocular foreign bodies.

The diagnostic criteria for OC were the presence of ophthalmoplegia, proptosis, chemosis, decreased visual acuity, or radiologic evidence of orbital inflammation or abscess (1, 9). The diagnosis of endophthalmitis was chiefly based on clinical manifestations, including the presence of corneal ulcers, hypopyon, anterior chamber cells, and inflammation in the vitreous body. Total corneal melting was defined as an ulcer or the dissolution of the entire cornea. The indications for evisceration were disfiguring blind eye, painful blind eye, uncontrolled endophthalmitis, or phthisis bulbi (15, 16). Representative images of these clinical features are shown in Figure 1.

### Procedures

The patient characteristics were recorded in an electronic medical record system. The demographic information included age and sex. Predisposing factors, such as trauma, sinusitis, dental infection, and previous history of surgery, were investigated. Data on systemic antibiotic and glucocorticoid administration, as well as surgical treatment, were recorded. Visual acuity, intraocular pressure (IOP), and orbital tension were also recorded. Visual acuity treatment was classified as NLP, light perception (LP), or better than LP. As the measurement of IOP was unavailable or performed *via* finger touch in many cases, this factor was not analyzed. The extent of the corneal ulcer was recorded and classified as no ulcer, local ulcer, or total corneal melting. If the condition was caused by trauma, the injury-causing objects, location of the ocular wound, and foreign bodies were recorded. For this analysis, the types of foreign bodies were identified and classified as metals, wood, or others. The location of the foreign bodies was classified as the anterior segment (anterior chamber and lens), posterior segment (vitreous body and retina), and orbit (outside of the eyeball but inside the orbit).

Samples from corneal scraping and aqueous or vitreous taps for culture were collected during surgery under anesthesia. The aqueous humor from the anterior chamber was aspirated through the corneal limbus using a needle on a 1-mL syringe. Vitreous specimens were collected before antibiotic injection or vitrectomy using a needle. The samples were inoculated for microorganism growth and antibiotic susceptibility testing according to the methods described in our previous study (13). Briefly, the samples were inoculated in trypticase soy broth (BACT/ALERT^®^ SA and BACT/ALERT^®^ SN, BioMerieux, Inc., Marcy-l’Étoile, France) overnight at 37°C. Subsequently, the broth was inoculated onto sheep blood agar and potato glucose agar for the growth of bacterial and fungal cultures, respectively. All bacterial isolates were subjected to species identification using an automated microbiological system, Vitek 2 Compact (BioMerieux, Inc., Marcy-l’Étoile, France), while all fungal isolates were identified by experienced technicians based on fungal morphology. Antibiotic susceptibility testing of the isolated bacteria was performed using Kirby-Bauer disc diffusion and minimal inhibitory concentration (MIC) methods, according to different antibiotics. Antibiotic susceptibility was determined according to the methods of the Clinical and Laboratory Standards Institute (CLSI).

Systemic antibiotics, including ceftazidime and levofloxacin, were intravenously administered to all patients as soon as OC was diagnosed. Intravenous glucocorticoids were administered in all cases except for those with suspected fungal infections.

### Statistical Analysis

All data were collected in an electronic database and cross-checked for errors. Statistical analyses were performed using SPSS for Windows, version 16.0 (SPSS, Inc., Chicago, Illinois, United States). Categorical variables were analyzed using χ^2^-tests. Continuous variables were evaluated for normality and the means were compared using two-tailed *t*-tests. Multiple logistic regression analysis was conducted to predict independent factors affecting evisceration. Statistical significance was set at *P* < 0.05.

## Results

### Demographic Characteristics

**FIGURE 2:**
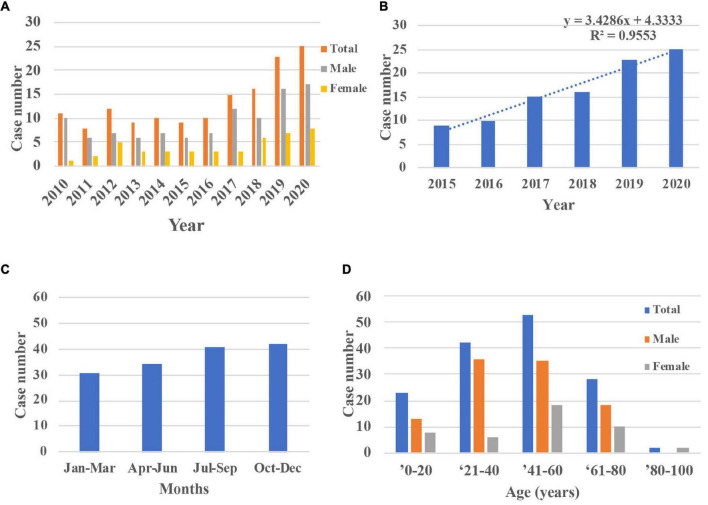
Demographic characters of inpatients with OC during 2010 and 2020. **(A,B)** The case numbers were similar in the first 5 years but increased in the last 5 years (*R* = 0.96). **(C)** The distributions across seasons did not differ significantly (*P* = 0.50). **(D)** The largest number of cases of OC occurred among patients aged 40–60 years and most patients were male. OC, orbital cellulitis.

This study included a total of 148 consecutive patients (148 eyes) admitted for OC. The average number of annual cases of OC was 13.45 ± 5.79 (Figure 2). The case numbers were similar in the first 5 years but increased significantly within the last 5 years (*R* = 0.96). The seasonal distributions did not differ significantly (*P* = 0.50). The mean patient age was 42.07 ± 20.35 years (range 1–84 years). The largest number of cases of OC (53 cases, 35.81%) occurred among patients aged 41–60 years, followed by those aged 21–40 years (42 cases, 28.38%). Most of the patients were men (104 cases, 70.27%).

### Clinical Characteristics

Regarding visual acuity measured at admission, half of the cases were NLP (74 cases, 50%), 22.30% were LP, and only 27.70% had better vision than LP (Table 1). Penetrating globe injury (77 cases, 52.03%) was the dominant cause of OC, followed by blunt ocular trauma (36 cases, 24.32%). In this study, 23 cases (16.22%) were caused by sinusitis, dental infection, and spread of skin inflammation and 12 cases (8.11%) without definite causation. IOFB were detected *via* radiographs or computed tomography (CT) scans in 43 cases (29.05%), most of which were iron (35 cases).

**TABLE 1:** Univariate analysis of the risk factors for NLP in patients with OC.

Factor	NLP no. (%)	LP or better no. (%)	*P*-value
Cases	74	74	
Age (years)			0.075
Sex			0.639
Male	54	50	
Female	20	24	
Penetrating trauma			0.007
Yes	47	30	
No	27	44	
Intraocular foreign body			0.048
Yes	27	16	
No	47	58	
Endophthalmitis			0.110
Yes	56	47	
No	18	27	
Corneal injury			0.000
Yes	49	30	
No	25	44	

*NLP, no light perception; OC, orbital cellulitis; LP, light perception.*

Endophthalmitis and total corneal dissolution were observed in 103 (69.59%) and 31 patients (20.95%), respectively. No cases of intracranial extension were observed. Most patients underwent evisceration (60 cases, 40.54%), followed by medical treatment alone (50 cases, 33.78%), pars plana vitrectomy (PPV, 23 cases, 15.54%), corneoscleral wound repair (11 cases, 7.43%), and drainage of orbital abscesses (4 cases, 2.70%).

### Risk Factors for No Light Perception

Owing to the high rate of NLP (50% at admission) in this study, the predisposing factors associated with NLP were analyzed. In univariate analysis, penetrating trauma, IOFB, and corneal injury differed significantly between patients with and without NLP (*P* = 0.007, *P* = 0.048, and *P* = 0.000, respectively), whereas age, sex, and endophthalmitis were not significantly different between the two groups (Table 1). Multivariate logistic regression analysis identified corneal injury as the highest risk factor for NLP (OR = 6.263, *P* = 0.000), penetrating trauma as a high-risk factor (OR = 2.972, *P* = 0.019), while IOFB was not associated with NLP (Table 2).

**TABLE 2:** Multivariate analysis of the independent risk factors for NLP.

Factor	*P*-values	OR	95% CI
Intraocular foreign body	0.866	1.087	0.412–2.867
Penetrating trauma	0.019	2.972	1.200–7.364
Corneal injury	0.000	6.263	2.236–17.540

*NLP, no light perception; OR, odds ratio; CI, confidence interval.*

### Risk Factors for Evisceration

As 60 patients (40.54%) underwent evisceration, it was important to explore the risk factors. In the univariate analysis, age, NLP, IOFB, endophthalmitis, and total corneal melting differed significantly between patients who did and did not undergo evisceration (*P* = 0.016, *P* < 0.0001, *P* = 0.002, *P* = 0.001, and *P* < 0.001, respectively). Sex and penetrating trauma did not differ significantly between the two groups (Table 3). Multivariate logistic regression analysis identified total corneal dissolution (OR = 83.019, *P* = 0.000) as the highest risk factor for evisceration, followed by NLP (OR = 5.425, *P* = 0.001) and IOFB (OR = 3.402, *P* = 0.016), while age (OR = 1.001, *P* = 0.686) and endophthalmitis (OR = 2.693, *P* = 0.099) were not associated with evisceration (Table 4).

**TABLE 3:** Univariate analysis of risk factors for evisceration in patients with OC.

Factor	Evisceration no. (%)	Non-evisceration no. (%)	*P*-value
Cases	60	88	
Age			0.016
Sex			0.501
Male	44	60	
Female	16	28	
Visual acuity			<0.0001
NLP	47	27	
LP or better	13	61	
Penetrating trauma			0.126
Yes	27	51	
No	33	37	
Intraocular foreign body			0.002
Yes	26	17	
No	34	71	
Endophthalmitis			0.001
Yes	57	46	
No	3	42	
Total corneal melting			<0.0001
Yes	30	1	
No	30	87	

*OC, orbital cellulitis; NLP, no light perception; LP, light perception.*

**TABLE 4:** Multivariate analysis of the independent risk factors for evisceration.

Factor	*P*-values	OR	95% CI
Age	0.686	1.006	0.979–1.033
Endophthalmitis	0.099	2.693	0.830–8.741
Intraocular foreign body	0.016	3.402	1.254–9.228
NLP	0.001	5.425	2.041–14.422
Total corneal dissolution	0.000	83.019	10.206–675.329

*OR, odds ratio; CI, confidence interval; NLP, no light perception.*

### Pathogen Characteristics

Microorganism detection showed positive results for 48 of 100 cases, including 23 cases (47.92%) with gram-positive bacteria, 18 cases (37.50%) with gram-negative bacteria, and 7 cases (14.58%) with fungi (Table 5). *Pseudomonas aeruginosa* and *Bacillus cereus* were the leading pathogens (nine cases each), followed by *Aspergillus* spp. (five cases), *Enterobacter cloacae* (four cases), and *Streptococcus* (three cases). Levofloxacin was the most effective antibiotic against Gram-positive bacteria. Vancomycin was effective against Gram-positive cocci, while cefepime and ceftazidime were effective against Gram-negative bacteria. Penicillin resistance was present in most gram-positive bacteria, cefuroxime resistance was present in Gram-positive bacilli, and ampicillin resistance was present in Gram-negative bacilli.

**TABLE 5:** Microbiological profile of OC.

Pathogen	Case number	Sensitive antibiotics in common	Resistant antibiotics in common
Gram-positive cocci	9		
*Streptococcus*	3	Levofloxacin, vancomycin, ofloxacin, chloramphenicol	Penicillin, tetracycline
*Staphylococcus aureus*	2	Levofloxacin, vancomycin, Ciprofloxacin, Moxifloxacin	Penicillin, oxacillin
*Staphylococcus epidermidis*	2	Levofloxacin, vancomycin, moxifloxacin, nitrofurantoin	None
Other Gram-positive cocci	2	NA	NA
Gram-positive bacilli	13		
*Bacillus cereus*	9	Levofloxacin, ofloxacin, amikacin	Penicillin, cefuroxime
*Bacillus subtilis*	2	Levofloxacin, ofloxacin, tobramycin, chloramphenicol	Cefuroxime, cefazolin, ceftazidime
Other Gram-positive bacilli	2	NA	NA
Gram-negative bacilli	17		
*Pseudomonas aeruginosa*	9	Levofloxacin, cefepime, ceftazidime, gentamicin	Ampicillin, sulbactam, nitrofurantoin, cefuroxime
*Enterobacter cloacae*	4	Levofloxacin, cefepime, ceftazidime, ciprofloxacin	Ampicillin, sulbactam, cefotetan, cefazolin
*Klebsiella pneumoniae*	2	Levofloxacin, cefepime, ceftazidime, gentamicin	Ampicillin
Other Gram-negative bacilli	2	NA	NA
Fungus	7		
*Aspergillus* spp.	5	None	None
Other fungus	2	NA	NA
Other pathogen	2	NA	NA

*OC, orbital cellulitis.*

## Discussion

Most previous studies on OC were conducted in Western countries, where sinus infection has been implicated as the major cause of OC. Children are the most susceptible population, with relatively good prognosis in vision and abscess drainage as the predominant surgery (1, 10, 17–19). In contrast, studies in East Asia have rarely been reported. The data in the current study showed that most patients with OC were adult men, that penetrating globe injury was the major cause, that two-thirds of the patients had NLP at discharge, and that evisceration was required in a large proportion of patients with OC.

Middle-aged men are the most susceptible to trauma-dominant OC. Age is an important predisposing factor for OC development. Children and early adolescents are populations reportedly most susceptible to OC, with an average case age of 19.92–25.7 years (1). In contrast, the present study included few patients (15.54%) under 20 years of age, with most patients (73.33%) aged 20–60 years. While the sex distribution is usually equal in patients with OC, a male preponderance has also been reported in studies from India, the US, and Canada (66, 73, and 74%, respectively), which is consistent with our findings (70.27%) (20–22). The high proportion of male patients may be attributed to the prevalence of work accidents as an etiological factor.

NLP is extremely common in trauma-dominant OC. With the development of antibiotics and surgical treatment, complete loss of vision due to OC is rare and the rate of blindness has decreased from 15–20 to 2.5–4.3% (1, 9, 23, 24). In contrast, 67.57% of the patients at discharge had permanent blindness. The severe loss of vision may be attributed to trauma and intraocular inflammatory or toxic factors, as endophthalmitis accounted for 68.92% of cases in the current study. Other potential mechanisms of visual loss include septic optic neuritis and thrombotic lesions in the vascular supply to the retina or optic nerve (25–27). In this study, penetrating trauma, corneal injury, and IOFB were identified as significant risk factors for NLP.

Medical treatment was effective in 33.78% of trauma-dominant OC inpatients, while evisceration accounted for 40.54% of the patients. Orbital cellulitis is an acute and aggressive disease requiring immediate and effective treatment. While working criteria for the surgical drainage of subperiosteal abscesses have been established, they may not be suitable for trauma-dominant OC (28–30). In the current study, intravenous antibiotics and glucocorticoids were administered to all cases as soon as OC was diagnosed, except in cases of suspected fungal infection. However, medical treatment was effective in only one-third of inpatients with trauma-dominant OC. PPV was performed when severe endophthalmitis developed and the fundus was visible, whereas evisceration was inevitable in eyes with total corneal melting. To our knowledge, no study has reported the risk factors associated with OC-related evisceration. The reported incidence rate of infectious keratitis leading to evisceration is 1.8% among patients with microbial keratitis in the US, and increases to 7–15% in the elderly population (31, 32). However, in the case of total corneal melting, the rate of evisceration increased to 35.4%, consistent with the current findings (32). We previously identified IOFB as an important risk factor for endophthalmitis and endophthalmitis as a significant factor for eviscerations (11–14). Together, these results suggest that total corneal melting, NLP, and IOFB are risk factors for evisceration in patients with OC. However, further investigations with larger sample sizes are required to confirm our observations.

Our results must be considered in light of several limitations. First, the study was limited by its retrospective nature; important data were not well recorded, including ocular proptosis and movement. Second, a relatively small proportion of patients underwent microorganism detection (100 of 148 cases, 67.56%), with a low positive rate (48 of 100 cases, 48%). Third, this study included only inpatients and outpatient data were unclear. Nonetheless, with a moderately large population of patients with OC, the results of the current study revealed the clinical characteristics of trauma-dominant OC and identified the risk factors for evisceration.

## Conclusion

Among hospitalized trauma-dominant patients with OC, middle-aged men were predominant and penetrating globe injury was the major cause. Significant complications such as complete visual loss and evisceration were unavoidable in many patients with OC in the current study. NLP, IOFB, and total corneal melting were the risk factors for evisceration. Therefore, better prevention and management strategies are urgently required.

## Data Availability Statement

The raw data supporting the conclusions of this article will be made available by the authors, without undue reservation.

## Ethics Statement

The studies involving human participants were reviewed and approved by the Medical Ethics Committee of Zhongshan Ophthalmic Center, Sun Yat-sen University. Written informed consent to participate in this study was provided by the participants’ legal guardian/next of kin. Written informed consent was obtained from the individual(s) for the publication of any potentially identifiable images or data included in this article.

## Author Contributions

FD and XL conceived, designed the study, and critically reviewed the manuscript. ZJ, XZ, XD, and YY participated in data collection and interpretation. XD contributed to the statistical analysis of the data. ZJ and XZ analyzed the data and wrote the first draft of the manuscript. All authors approved the submitted version.

## Conflict of Interest

The authors declare that the research was conducted in the absence of any commercial or financial relationships that could be construed as a potential conflict of interest.

## Publisher’s Note

All claims expressed in this article are solely those of the authors and do not necessarily represent those of their affiliated organizations, or those of the publisher, the editors and the reviewers. Any product that may be evaluated in this article, or claim that may be made by its manufacturer, is not guaranteed or endorsed by the publisher.
